# Nonpharmacologic and Nonsurgical Weight Management Interventions for Patients With Advanced CKD: A Scoping Review of the Medical Literature

**DOI:** 10.1016/j.xkme.2025.101004

**Published:** 2025-04-15

**Authors:** Kamel Omer, Kristin K. Clemens, Yunxu Zhu, Heather LaPier, Louise Moist, Jaclyn Ernst, Sonja M. Reichert, Alla Iansavichene, Michael Chiu

**Affiliations:** 1 Schulich School of Medicine and Dentistry, Western University, London, Ontario, Canada; 2 Division of Endocrinology and Metabolism, Department of Medicine, Western University, London, Ontario, Canada; 3 Centre for Diabetes, Endocrinology and Metabolism, St. Joseph’s Health Care London, London, ON, Canada; 4 ICES, Toronto, Ontario, Canada; 5 Lawson Research Institute, London, Ontario, Canada; 6 Centre for Quality, Innovation and Safety, Western University, London, Ontario, Canada; 7 Division of Nephrology, Department of Medicine, Western University, and London Health Sciences Centre, London, Ontario, Canada; 8 Kidney Clinical Research Unit, London Health Sciences Centre, London, Ontario, Canada; 9 Division of General Internal Medicine, Department of Medicine, Western University, and London Health Sciences Centre, London, Ontario, Canada; 10 Department of Family Medicine, Western University, London, Ontario, Canada; 11 Corporate Academics, Health Sciences Library, London Health Sciences Centre, London, Ontario, Canada

**Keywords:** Body mass index, chronic kidney disease, diet, end-stage kidney disease, exercise, obesity, weight loss

## Abstract

**Rationale & Objective:**

Obesity is associated with morbidity and mortality in people with chronic kidney disease (CKD). Identifying safe and effective nonpharmacologic and nonsurgical interventions to achieve a healthier body weight is essential.

**Study Design:**

Scoping review of observational studies and randomized control trials.

**Setting & Study Populations:**

Adults aged ≥18 years with a body mass index (BMI) ≥30 kg/m^2^ and advanced CKD (category G3-G5D).

**Selection Criteria for Studies:**

Following the Preferred Reporting Items for Systematic Reviews and Meta-analyses Extension for Scoping Reviews (PRISMA-ScR), we systematically searched 2 electronic databases (MEDLINE and Embase) for studies that examined the effect of nonpharmacologic and nonsurgical interventions for weight loss between January 2010-July 2024. Outcomes included weight loss and BMI. We also examined adherence, whether participants were involved in the design of the study, and adverse events.

**Data Extraction:**

Two reviewers screened relevant citations and extracted study characteristics and outcomes. Discrepancies were resolved by a third reviewer.

**Analytical Approach:**

Study data were summarized descriptively following guidance from the PRISMA-ScR.

**Results:**

Of the 2,453 citations, 17 met inclusion criteria (9 randomized controlled trials, 2 nonrandomized trials, 5 prospective cohort studies, and 1 retrospective cohort study) and included a total of 960 participants. Interventions included exercise programs, dietary therapy, and/or cognitive behavioral therapy with follow-up ranging from 3-12 months. It appeared that dietary intervention that promoted significant caloric restriction over the short term led to the most weight loss (average, 7 kg). Interventions with monitored coaching appeared helpful. No adverse events were reported. None of the studies involved participants as partners.

**Limitations:**

Not all studies included participants’ estimated glomerular filtration rate or BMI category, and we may have included some without severe CKD or BMI ≥30 kg/m^2^.

**Conclusions:**

Programs encouraging very low-energy diets along with monitored coaching, may result in modest short-term weight loss. Patient views on these programs and their longer term success remain unclear.

Obesity, conventionally defined by a body mass index (BMI) ≥30 kg/m^2^, affects over 50% of people with chronic kidney disease (CKD).^1^ Obesity has been associated with worsening kidney function^2^ and development of kidney failure, requiring kidney replacement therapy.^3^ It is also a major barrier to access a life-saving kidney transplant given its association with peri- and postsurgical morbidity and mortality.^4^^,^^5^

Despite the magnitude of this problem, providers have little guidance on how to counsel patients with CKD (particularly advanced and end-stage CKD) to lose weight. This is in part because of limited research on effective strategies for weight management. Randomized controlled trials (RCTs) of nutritional intervention,^6^ exercise,^7^ surgery,^8^^,^^9^ or combination therapy have been generally limited to those with preserved kidney function.^10^, ^11^, ^12^ Medications such as glucagon-like peptide-1 agonists have demonstrated efficacy and safety for preserving kidney function, reducing cardiovascular outcomes, and an additional benefit of weight loss in patients with CKD.^13^

Scoping reviews help to rapidly identify and synthesize the breadth and depth of available evidence. The present review is an essential step toward understanding how to care for patients with obesity, informing clinical practice guidelines, and identifying gaps for future clinical trials to help patients with advanced CKD achieve a healthy weight. Although there have been systematic and narrative reviews of observational studies that focus on the benefits of pharmacologic therapy for weight loss in advanced CKD (category G3-5D),^14^ combination pharmacologic and nonpharmacologic care is often the desired approach. However, there are also some people who prefer completely nonpharmacologic and nonsurgical interventions (or they may have side effects or contraindications to pharmacotherapy). A thoughtful and thorough review is essential because people with advanced CKD have restrictive nutritional requirements (low potassium and low phosphate diets), functional limitations, and/or multiple comorbid conditions such as diabetes and heart disease, which make finding safe nutritional and exercise interventions challenging. The primary aim of this scoping review is to summarize the published literature on the efficacy of nonpharmacologic and nonsurgical weight management interventions in people with advanced CKD and obesity.

## Methods

This scoping review was designed using the Preferred Reporting Items for Systematic Reviews and Meta-analyses (PRISMA) Extension for Scoping Reviews Extension for Scoping Reviews guidelines,^15^ which are adapted from the 2020 PRISMA guidelines.^16^

### Population

We included English language studies published between 2010 until the end of July 2024 of adults aged ≥18 years with category 3-5D CKD and obesity defined by BMI of ≥30 kg/m^2^. Inclusion and exclusion criteria are described in Appendix A. Our database search strategy is described in Appendix B.

### Weight Loss Intervention

We included published studies that examined the efficacy of nonpharmacologic and nonsurgical weight loss strategies. Nonpharmacologic and nonsurgical interventions included nutritional interventions, exercise, and cognitive behavioral therapy (CBT).

### Study Designs

We included observational (cohort, case-control, and pre- and postintervention studies) and RCTs. We excluded studies written in languages other than English, animal experiments, case reports, case series, and review articles.

### Control

Studies could either have or not have a control group.

### Outcomes

Our primary outcome of interest was weight loss. Secondary outcomes included changes in measurements of body composition, such as waist circumference, percentage body fat, fat-free mass, BMI, and waist-to-hip ratio. We also examined adherence to interventions, safety, and participant engagement in the development of weight management interventions.

### Information Sources

An experienced clinical librarian (AI) designed a comprehensive search strategy based on our objectives, inclusion criteria, and outcomes. Two databases (MEDLINE and Embase) were systematically searched using relevant key words and Medical Subject Heading terms. Our search strategy is shown in Appendix B. Citations were imported into and maintained in Covidence.^17^

### Selection of Sources of Evidence

Abstracts and titles were screened in duplicate (KO, MC, and HL). After reviewers screened the first 25 citations, a kappa statistic was calculated to ensure inter-rater reliability. After the completion of citation screening, an independent reviewer (KC) resolved any discrepancies.

### Data Charting and Extraction

We developed a standardized data collection tool to abstract included articles. We collected study identifiers (origin, authors), weight loss interventions (eg, nutritional intervention, exercise, and CBT), and study outcomes. If data of interest were not available in the published report, we attempted to contact the corresponding author of the study for this information.

### Synthesis of Results

Given the heterogeneity of studies, we present results descriptively with means or medians and *P* values, if provided.

## Results

**Figure 1:**
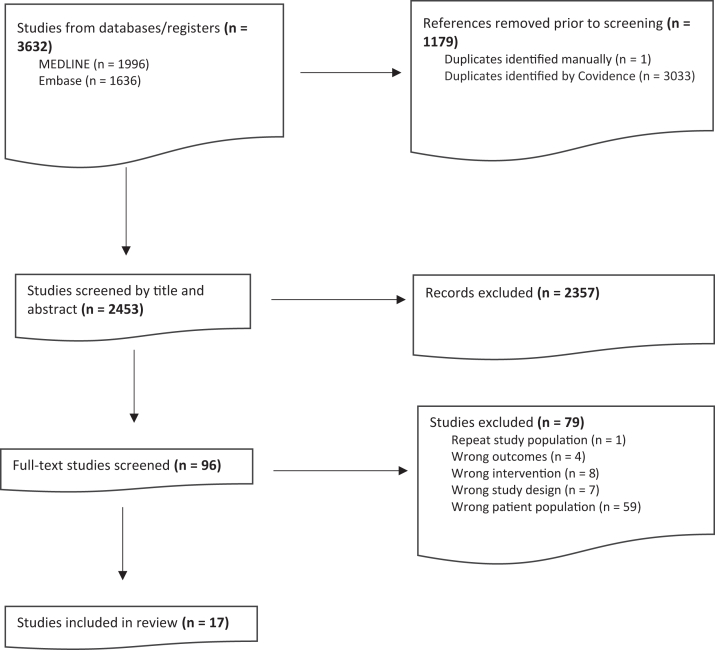
Flow diagram for included and excluded studies.

There were 3,632 records retrieved, and after deduplication, 2,453 unique citations were identified from the literature search. Of these, 17 studies met inclusion criteria, involving 960 participants with primarily stage 3-5 CKD, including dialysis. The selection of studies is summarized in Figure 1 and their characteristics are included in Table 1.^7^^,^^18^, ^19^, ^20^, ^21^, ^22^, ^23^, ^24^, ^25^, ^26^, ^27^, ^28^, ^29^, ^30^, ^31^, ^32^, ^33^ Seven studies were conducted in the United States, 4 in Brazil, 3 in Australia, 1 in Canada, 1 in China, and 1 in India. There were 9 RCTs, 2 nonrandomized trials, and 6 observational cohort studies. Study follow-ups ranged from 3 to 12 months.

**Table 1:** Details of the 13 Included Studies, Along With Weight Loss Intervention Descriptions

	Reference, Location, and Design	Participant Population	Number of Participants	Nutritional Intervention	Exercise Regimen	Relevant Outcomes Measured	Findings
**1**	Friedman et al^18^ (2013) USA Cohort study (pre-post analysis) 12 wk follow-up	CKD 3-5	6	Very low-calorie ketogenic diet: 800 kcal/d with at least 75 g protein and all essential vitamins and nutrients Carbohydrate consumption was restricted to <50 g/d	Not supervised Combination of aerobic and resistance training with a goal of burning at least 2000 cal/wk	Body weight BMI Lean body mass (%) % body fat	Body weight (min, max): 118.5 (94.8, 140.0) kg pre-intervention to 104.3 (85.0, 115.8) kg post-intervention; *P* = 0.03 BMI (min, max): 38.6 (32.8, 43.7) kg/m^2^ pre-intervention to 34.0 (29.6, 37) kg/m^2^ post-intervention; *P* = 0.03 Lean body mass (min, max): 57.6% (54.7%, 71.4%) pre-intervention to 64% (62.1%, 75.9%) post-intervention; *P* = 0.11 Fat mass (min, max): 42.4% (28.6%, 45.3%) pre-intervention to 36% (24.1%, 38.8%) post-intervention; *P* = 0.11
**2**	Ikizler et al^19^ (2018) USA Randomized controlled trial 4 mo follow-up 2 × 2 factorial design: 4 comparison groups: (1) caloric restriction and aerobic exercise, (2) caloric restriction alone, (3) aerobic exercise alone, (4) usual care	CKD 3-5	111	Caloric restriction: decrease daily caloric intake by 10%-15% All participants asked to avoid a high-calorie diet and were provided dietary recommendations	Supervised 30-45 min of aerobic exercise 3×/week for 4 mo at center Training intensity individualized and gradually increased as directed by certified exercise specialists	Body weight BMI WHR % body fat	Diet and exercise significantly improved body weight, BMI, and % body fat: Mean treatment effect (CI): body weight −2.39 (−4.27 to −0.52) kg, *P* = 0.016; BMI −0.83 (−1.51 to −0.14) kg/m^2^, *P* = 0.025; body fat % −0.52 (−1.88 to 0.84), *P* = 0.015 Diet significantly improved body weight, BMI, and % body fat: Mean treatment effect (CI): body weight −1.80 (−3.62 to 0.01) kg, *P* = 0.004; BMI −0.55 (−1.22 to 0.12) kg/m^2^, *P* = 0.009; body fat −1.58% (−2.89% to −0.028%), *P* = 0.021 Exercise alone and usual care did not significantly affect weight loss parameters
**3**	Malin et al^20^ (2020) USA Cohort study (pre-post analysis) 3 mo follow-up	CKD 3-5	8	Registered dietitian developed individualized 500-kcal hypocaloric diets with 50%-60% carbohydrate, 25%-30% fat, and 15% (ie, 0.8-1.0 g/kg/d) protein	Increasing aerobic activity with supervision by nurse or exercise physiotherapist Treadmill 5 d/wk for 30-60 min/d at 65%-85% of maximum HR Breakdown: 30-40 min/d for wk 1-4, then 40-50 min/d for wk 5-8, then 50-60 min/d until wk 12	Body weight BMI % body fat Fat-free mass	Body weight (± SEM) 122.6 ± 7.0 kg pre-intervention to 117.3 ± 6.3 kg post-intervention; *P* = 0.001 BMI (± SEM) 43.7 ± 2.1 kg/m^2^ pre-intervention to 41.8 ± 1.9 kg/m^2^ post-intervention; *P* < 0.001 % body fat (± SEM) 48.5% ± 3.2% pre-intervention to 46.2% ± 3.1% post-intervention; *P* = 0.001 Fat-free mass (± SEM) 63.2 ± 3.9 kg pre-intervention to 63.4 ± 4.1 kg post-intervention, *P* = 0.74
**4**	Patil et al^21^ (2013) India Prospective randomized clinical trial 6 mo follow-up in group with life-style intervention	Diabetic nephropathy (proteinuria)	76	Dietitian-supported decrease in caloric intake by 500-1000 kcal/d, with nutritional education	Exercise advice was suggested and provided by dietitian: 30 min of moderate-intensity physical activity most days of week. Regular check-ins with participants.	BMI WHR	BMI (± SD) 33.01 ± 4.01 kg/m^2^ pre-intervention to 31.06 ± 3.53 kg/m^2^ post-intervention; mean treatment effect −1.95 ± 1.10 kg/m^2^, *P* = 0.00 WHR (± SD) 1.03 ± 0.06 pre-intervention 1.00 ± 0.06 post-intervention; mean treatment effect −0.03 ± 0.02, *P* = 0.00
**5**	Hajjar et al^22^ (2022) Canada Cohort study (pre-post analysis) 12 mo follow-up	CKD 4-5	80	Encouragement of healthy eating habits	Not supervised Encouraged daily exercise with gradual increase in effort and time: initially for 10 to 15 min/d	Body weight BMI	Mean change in body weight at 1 y (IQR): −3.4 (−6.8 to 0.7) kg Change in BMI at 1 y (IQR): −1.6 (−3.5 to 0.6) kg/m^2^
**6**	Cheng et al^23^ (2022) Australia Cohort study (pre-post analysis) Retrospective analysis of medical charts 34 wk follow-up	CKD 4-5 (*n* = 3) or dialysis patients (*n* = 11)	14	VLED (not described)	N/A	Body weight	Median max weight loss from VLED (IQR) over 34 wk was 13.7 (6.0-20.4) kg
**7**	Pereira et al^24^ (2023) Brazil Nonrandomized clinical trial 6 mo follow-up	CKD 4-5	23	Coaching of dietary intake by renal dieticians	N/A	BMI WHR	BMI (IQR): 32.6 (30.2-39.3) kg/m^2^ pre-intervention to 32.6 (29.4-37.3) kg/m^2^ post-intervention; *P* = 0.976 WHR (IQR): 0.95 (0.92-0.99) m pre-intervention to 0.97 (0.92-1.0) m post-intervention; *P* = 0.158
**8**	Lao et al^25^ (2023) China Nonrandomized clinical trial 3 mo follow-up	CKD 3-4	28	Time-restricted feeding (TRF): consume 3 meals within 8 h. No calorie-containing food could be consumed during fasting period.	N/A	Body weight BMI % body fat Lean body mass WC	Body weight (± SD) TRF vs. control: -2.8 ± 2.9 kg vs −0.4 ± 1.4 kg; *P* = 0.010 BMI (± SD) TRF vs control: −1.1 ± 1.1 kg/m^2^ vs −0.2 ± 0.6 kg/m^2^; *P* = 0.013 % body fat (± SD) TRF vs control: −1.0% ± 2.0% vs −0.5% ± 2.5%; *P* = 0.592 Fat-free mass (± SD) TRF vs control: −1.1 ± 1.4 kg vs 0.0 ± 1.6 kg; *P* = 0.066 WC (± SD) TRF vs control: −1.9 ± 3.2 cm vs +0.1 ± 3.1 cm; *P* = 0.110
**9**	Baria^7^ (2014) Brazil Randomized controlled trial Home-based exercise vs center-based exercise vs control 12 wk follow-up	CKD 3-4	29	No nutritional intervention Renal-specific diet: 25-30 kcal/adjusted body weight/d and 0.6-0.8 g/adjusted body weight/d of protein	Center-based exercise: aerobic training on treadmill 3×/wk during 12 wk every other day under supervision of an exercise physiologist Home-based exercise: aerobic training at home, 3×/wk every other day according to instructions given by exercise physiologist. Included 3 supervised visits, then weekly telephone follow-up	Body weight BMI Fat mass Lean body mass WC	Center-based body weight (± SD): 86.2 ± 19.4 kg pre-intervention vs 86.1 ± 20.7 kg post-intervention; *P* = 0.6 *P* interaction with control = 0.12 Home-based body weight (± SD): 90.9 ± 12.4 kg pre-intervention vs 89.3 ± 11.9 kg post-intervention; *P* = 0.08 *P* interaction with control < 0.01 Control body weight (± SD): 84.8 ± 7.8 kg pre-intervention vs 85.9 ± 7.7 kg post-intervention; *P* = 0.05 Center-based BMI (± SD): 30.8 ± 5.1 kg/m^2^ pre-intervention vs 30.7 ± 5.5 kg/m^2^ post-intervention; *P* = 0.75 *P* interaction with control = 0.18 Home-based BMI (± SD): 30.9 ± 3.9 pre-intervention kg/m^2^ vs 30.4 ± 3.8 kg/m^2^ post-intervention; *P* = 0.08 *P* interaction with control < 0.01 Control BMI (± SD) 29.6 kg/m^2^ ± 1.9 pre-intervention vs 30.0 kg/m^2^ ± 1.8 post-intervention; *P* = 0.05 Center-based total body fat (± SD): 24.2 ± 5.1 kg pre-intervention vs 23.4 ± 5.8 kg post-intervention; *P* = 0.18 *P* interaction with control = 0.05 Home-based total body fat (± SD): 29.0 ±6.0 kg pre-intervention vs 28.4 ± 6.3 kg post-intervention; *P* = 0.14 *P* interaction with control = 0.02 Control total body fat (± SD): 24.3 ± 4.8 kg pre-intervention vs 24.7 ± 5.1 kg post-intervention; *P* = 0.09 Center-based lean body mass (± SD): 52.5 ± 5.4 kg pre-intervention vs 53.7 ± 5.6 kg post-intervention; *P* < 0.01 *P* interaction with control = 0.06 Home-based lean body mass (± SD): 60.5 ± 7.5 kg pre-intervention vs 60.5 ± 6.0 kg post-intervention; *P* = 0.96 *P* interaction with control = 0.79 Control lean body mass (± SD): 58.2 ± 7.4 kg pre-intervention vs 58.3 ± 7.3 kg post-intervention; *P* = 0.68) Center-based WC (± SD): 106.8 ± 16.7 cm pre-intervention vs 104.9 ± 16.0 cm; *P* = 0.03 *P* interaction with control = 0.01 Home-based WC (± SD): 108.3 ± 8.7 cm pre-intervention vs 106.9 ± 7.8 cm post-intervention; *P* = 0.09 *P* interaction with control = 0.04 Control WC (± SD): 101.8 ± 6.0 cm pre-intervention vs 103.3 ± 5.7 cm post-intervention; *P* = 0.16
**10**	Aoike et al^26^ (2015) Brazil Randomized controlled trial Home-based exercise vs control 12 wk follow-up	CKD 3-4	29	No nutritional intervention Renal-specific diet containing 30 kcal/kg/d and 0.6-0.8 g/kg/d of protein	Home-based moderate-intensity exercise program Monitored weekly by telephone calls and monthly by individual visits to support and assess progress and adherence Patients recorded their average HR and perceived exertion using Borg scale Aerobic training consisted of walking near home 3×/wk for 30 min every other day. Duration increased by 10 min every 4 wk until wk 8	Body weight BMI % body fat Lean body mass	Body weight (± SD): Home-based: 83.0 ± 14.0 kg pre-intervention to 82.3 ± 13 kg post-intervention Control: 84.2 ± 11.3 kg pre-intervention to 84.4 ± 11.4 kg post-intervention; *P* interaction = 0.328 BMI (± SD): Home-based: 31.7 ± 4.5 kg/m^2^ pre-intervention to 31.4 ± 4.5 kg/m^2^ post-intervention Control: 30.7 ± 4.1 kg/m^2^ pre-intervention to 30.7 ± 4.0 kg/m^2^ post-intervention; *P* interaction = 0.385 % body fat (± SD): Home-based: 38.4% ± 8.6% pre-intervention to 39.0% ± 10.5% post-intervention Control: 34.4% ± 9.6% pre-intervention to 34.3% ± 10.2% post-intervention; *P* interaction = 0.481 Lean body mass (± SD): Home-based: 48.6 ± 11.5 kg pre-intervention to 48.3 ± 11.1 kg post-intervention Control: 50.5 ± 9.1 kg pre-intervention to 50.0 ± 9.0 kg post-intervention; *P* interaction = 0.766
**11**	Aoike et al^27^ (2018) Brazil Randomized controlled trial Home-based exercise vs center-based exercise vs control 24 wk follow-up	CKD 3-4	40	No nutritional intervention Renal-specific diet containing 30 kcal/kg/d and 0.6-0.8 g/kg/d of protein	Moderate aerobic exercise Home-based: Monitored weekly by telephone calls and monthly by individual visits to support and assess progress and adherence Patients recorded their average HR and perceived exertion using Borg scale Aerobic training consisted of walking near home 3×/wk for 30 min every other day Center-based: Aerobic exercise program performed at an exercise center on a treadmill 3×/wk on every other day under the supervision of an exercise physiologist	BMI	Home-based exercise BMI (± SD): 31.1 ± 4.6 kg/m^2^ pre-intervention to 30.4 ± 3.4 kg/m^2^ post-intervention Center-based exercise BMI (± SD): 31.8 ± 4.5 kg/m^2^ pre-intervention to 31.2 ± 5.2 kg/m^2^ post-intervention Control BMI (± SD): 30.7 ± 4.1 kg/m^2^ pre-intervention to 31.5 ± 4.8 kg/m^2^ post-intervention
**12**	Sheshadri et al^28^ (2020) USA Randomized controlled trial 6 mo follow-up	Dialysis patients	54	N/A	Pedometers provided to intervention group Group received weekly counseling to encourage increasing step count by 10% compared to previous week	BMI Fat mass	BMI change (CI) intervention vs control at 6 mo: −1.0 (−1.8 to −0.2) kg/m^2^; *P* < 0.01 Fat mass change (CI) intervention vs control at 6 months: −4.3 (−7.1 to −1.5) kg; *P* < 0.01
**13**	Lyden et al^29^ (2021) USA Randomized controlled trial 24 wk follow-up	CKD 2 (with ACR >30 mg/g) CKD 3-4	106	N/A	SLIMM (Sit Less, Interact, Move More) intervention Participants given feedback on most sedentary periods and instructed to engage in light-intensity activities once per hour Control: Participants encouraged to engage in >150 min/wk of exercise	BMI % body fat WC	BMI (CI) between-group difference: −1.1 (−1.9 to −0.3) kg/m^2^ % body fat (CI) between-group difference: −2.1% (−4.4% to −0.2%) WC (CI) between-group difference: −0.4 (−3.1 to 0.9) cm
**14**	Howden et al^30^ (2013) Australia Randomized controlled trial 12 mo follow-up	CKD 3-4	90	No specific nutritional restrictions. Dietitian-led life-style modification focusing on sustainable diet to assist with weight loss.	Supervised exercise by clinical exercise physiologist 150 min of moderate-intensity exercise per week Combination of 20-30 min of aerobic activity followed by whole-body resistance training with machines, free weights, resistance bands, and Swiss ball	Body weight BMI WC	Body weight change (± SD) intervention vs control: −1.8 ± 4.2 kg vs +0.7 ± 3.7 kg; *P* = 0.01 BMI change (± SD) intervention vs control: -0.6 ± 1.4 kg/m^2^ vs +0.3 ± 1.4 kg/m^2^; P = 0.01 Waist circumference (± SD) intervention vs control: −1.4 ± 5.5 cm vs +1.6 ±5.0 cm; *P* = 0.01
**15**	St-Jules et al^31^ (2023) USA Randomized controlled trial 6 mo follow-up 2 × 2 factorial design: (1) ADVICE, (2) MONITORING, (3) SCT, (4) COMBINED	CKD 2-4	186	Participants given energy intake goal of 500 kcal/d deficit	150 min/wk of moderate-intensity physical activity	Body weight	Body weight change (± SD): ADVICE −1.2 ± 4.3 kg MONITORING −2.3 ± 3.4 kg SCT −1.4 ± 3.1 kg COMBINED −2.7 ± 4.4 kg
**16**	Navaneethan et al^32^ (2015) USA Cohort study (pre-post analysis) 3 mo follow-up	CKD 3-5	9	Participants given energy intake goal of 500 kcal/d deficit There were weekly, in-person check-ins	Supervision by exercise physiologist or research nurse Aerobic exercise 5 d/wk to achieve 65%-85% max HR for 45-60 min/session.	Body weight BMI Body fat % Fat mass WC	Body weight change (IQR) at 3 mo: −5.1 (−6.5 to −4.1) kg; *P* = 0.004 BMI change (IQR) at 3 mo: −1.9 (−2.6 to −1.4) kg/m^2^; *P* = 0.004 Body fat % change (IQR) at 3 mo: −1.8% (−2.6% to −1.6%); *P* = 0.004 Fat mass change (IQR) at 3 mo: −4.9 (−5.9 to −3.0) kg; *P* = 0.04 Waist circumference change (IQR) at 3 mo: −3.0 (−8.3 to −2.3) cm; *P* = 0.004
**17**	Lassemillante et al^33^ (2016) Australia Cohort study (pre-post analysis) 12 mo follow-up	Dialysis patients	5	Modified low-calorie diet with average of 950 kcal/d and 100 g protein/d using Optifast meal replacement shakes and bars	None	Body weight	Median of 7% body weight lost (range, 5.2-11.4)

Abbreviations: ACR, albumin-to-creatinine ratio; BMI, body mass index; CI, confidence interval; CKD, chronic kidney disease; HR, heart rate; IQR, interquartile range; N/A, not applicable; SCT, social cognitive theory; SD, standard deviation; SEM, standard error of the mean; TRF, time-restricted feeding; VLED, very low-energy diet; WC, waist circumference; WHR, waist-to-hip ratio.

We contacted the corresponding authors to gain additional information about the primary outcomes of our study but did not receive responses.

### Interventions

Five studies examined the utility of a nutritional intervention, 7 studied the utility of exercise, and 5 included a combination of nutritional interventions and exercise. CBT was used in 3 studies.

### Description of Nutritional Interventions

Nutritional interventions primarily promoted caloric restriction. For example, the intervention used by Ikizler et al^19^ promoted calorie reduction by 10%-15% and the avoidance of high-calorie foods. In their study, Patil et al^21^ restricted calories to 500-1,000 kcal/d and promoted choosing smaller meal portions, more fruits and vegetables, and leaner meats. Cheng et al^23^ encouraged a very low-energy diet, but the details of the diet were not thoroughly described. St-Jules et al^31^ and Navaneethan et al^32^ required that participants achieve a 500 kcal/d energy deficit.

Other studies, including that of Friedman et al,^18^ recommended both caloric restriction and restriction of macronutrients (very low-calorie ketogenic diet: 800 kcal/d with 75 g protein and carbohydrates restricted to <50 g/d). Malin et al^20^ studied a dietitian-developed hypocaloric diet of 500 kcal/d, with a macronutrient ratio of 60:25:15 (carbohydrate:fat:protein). Lassemillante et al^33^ studied a modified low-calorie diet using meal replacements (Optifast) in which participants were restricted to an average of 950 kcal/d, which included 100 g protein/d.

Lao et al^25^ examined the efficacy of time-restricted eating. In this study, participants were required to consume all 3 meals within an 8-hour period (with an unspecified high-quality, low-protein diet). Participants were only allowed to consume non–calorie-containing food or beverages in the fasting period.

Three studies also promoted coaching and CBT for behavior change in their intervention. For example, Hajjar et al^22^ had nutritionists coach participants on healthy eating habits, such as eating 3 small meals per day, reducing carbohydrate intake, optimizing protein, reducing excess caloric intake, eliminating processed snacks, and using food diaries. Pereira et al^24^ used CBT techniques that had participants attend 15 weekly or biweekly sessions with a renal dietitian with a focus on building healthy eating habits, diet mentality, hunger and satiety, and body respect. St-Jules et al^31^ used CBT techniques grounded in social cognitive theory-based behavioral group counseling over 14 web-based group conferencing sessions led by registered dieticians.

### Description of Exercise Interventions

Exercise interventions differed in frequency, intensity, duration (time), and type of activity (frequency, intensity, time, and type). Aerobic exercise was studied most often (10 studies). Exercise focused on sustained and repetitive movement, using different modalities of movement and protocols. Nine studies included an exercise coach to promote physical activity among participants.

For example, walking was studied by Malin et al,^20^ who promoted treadmill walking 5 days per week for 30-60 minutes to achieve 65%-85% maximum heart rate. Baria et al^7^ and Aoike et al^26^ studied the effect of unsupervised exercise at home versus supervised treadmill walking in-center. Sheshadri et al^28^ encouraged walking with the use of pedometers in which participants were encouraged to increase their step count by 10% compared to the previous week.

In Lyden et al,^29^ participants in the Sit Less, Interact, Move More (SLIMM) intervention were provided feedback on sedentary periods using an accelerometer and instructed to engage in light-intensity activities at least once per hour to increase activity levels. The control group engaged in >150 min/wk of exercise. The intervention used Patil et al^21^ included 30 minutes of nonmonitored moderate-intensity physical activity per week of any type. In the study of Hajjar et al,^22^ participants were encouraged to undergo daily exercise with a gradual increase in effort and time, starting at 10-15 minutes per day. Ikizler et al^19^ had participants undergo 30-45 minutes of supervised aerobic exercise in-center 3 times per week with a gradual increase in intensity. St-Jules et al^31^ had participants participate in 150 minutes per week of moderate-intensity physical activity that was not supervised. Navaneethan et al^32^ had participants undergo supervised aerobic exercise 5 days per week to achieve 65%-85% of their maximum heart rate.

Resistance training, in addition to aerobic activity, was encouraged in 2 studies. Friedman et al^18^ encouraged participants to expend 2,000 calories per week through a combination of aerobic and resistance training, using weights and bands for the resistance training component. Physical activity was not monitored. Howden et al^30^ prescribed 150 minutes of moderate-intensity exercise per week. The exercise regimen included a combination of 20-30 minutes of aerobic activity, which was followed by whole-body resistance training with free weights, machines, or resistance bands. This activity was supervised by an exercise physiologist or research nurse.

### Efficacy of Interventions

#### Weight Loss and BMI

**Figure 2:**
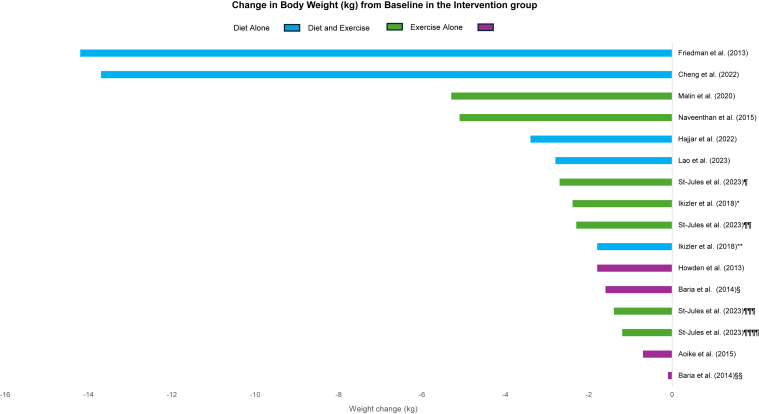
Changes in weight from baseline in the intervention groups. Summary of absolute weight change (kilograms) from baseline in each of the included studies. Groups in Ikizler et al^19^ (2018), Baria et al^7^ (2014), and St-Jules et al^31^ (2023) reported separately. ∗ = diet and exercise arm, ∗∗ = diet alone arm; § = home-based exercise, §§ = center-based exercise; ¶ = combined education/group counseling/tech-based self-monitoring, ¶¶ = education/tech-based self-monitoring, ¶¶¶ = education/group counseling, ¶¶¶¶ = education alone.

**Figure 3:**
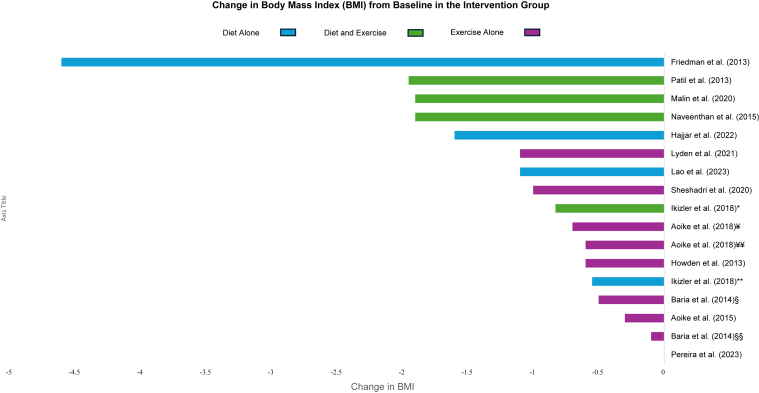
Changes in BMI from baseline in the intervention groups. Summary of the absolute BMI change from baseline in each of the included studies. Groups in Ikizler et al^19^ (2018), Aoike et al^26^ (2015), and Baria et al^7^ (2014) reported separately. ∗ = diet and exercise arm, ¥ = home-based exercise, ¥¥ = center-based exercise; ∗∗ = diet alone arm; § = home-based exercise, §§ = center-based exercise.

Weight loss was examined as an outcome in 12 studies and BMI in 13 studies. Changes in weight and BMI from baseline in the intervention group are summarized in Figures 2 and 3. The relationship between the initial BMI and the total amount of weight loss is summarized in Figure S1.

It appeared that intensive dietary interventions promoted the most weight loss and reduction in BMI. For example, the very low-calorie ketogenic diet of Friedman et al^18^ and promotion of exercise to burn 2,000 calories per week was associated with a weight loss of 14.2 kg (*P* = 0.03) and a BMI change of −4.6 kg/m^2^ (*P* = 0.03). The very low-calorie diet used by Cheng et al^23^ was associated with a median maximum weight loss of 13.7 kg.

In contrast, interventions that promoted healthier eating habits, such as the retrospective cohort study of Hajjar et al^22^ (eating 3 small meals, reducing additional carbohydrate calories, and limiting snacking, with exercise) had more subtle effects on weight and BMI (mean change of −3.4 kg and −1.6 kg/m^2^ at 1 year). The prospective cohort study of Lao et al^25^ used time-restricted feeding and described a mean decrease in body weight of −2.8 kg (*P* = 0.01) and change in BMI of −1.1 kg/m^2^ (*P* = 0.01) between the 2 groups. Ikizler et al^19^ studied the effects of caloric restriction in addition to avoiding high-calorie foods without exercise and noted weight loss of −1.8 kg (*P* = 0.004) and BMI change of −0.55 kg/m^2^ (*P* = 0.01). In a prospective cohort study, Pereira et al^24^ used CBT techniques that had dieticians coach participants on healthy eating habits and did not demonstrate any change in BMI. Lassemillante et al^33^ studied the use of meal replacements that led to weight losses of 7%.

The independent effect of exercise on weight loss and BMI was examined in 5 studies with modest effects. The RCT conducted by Baria et al^7^ compared home-based aerobic exercise versus center-based aerobic exercise versus usual care. In this study, the home-based aerobic exercise group had −1.6 kg of weight loss (*P* = 0.08) and a BMI change of −0.5 kg/m^2^ (*P* = 0.08), the center-based aerobic exercise group had −0.1 kg of weight loss (*P* = 0.6) and a BMI change of −0.1 kg/m^2^ (*P* = 0.75), and the usual care group had +1.1 kg weight gain (*P* = 0.05) and BMI change of +0.4 kg/m^2^ (*P* = 0.05). Howden et al^30^ examined the effects of supervised exercise and described −1.8 kg of weight loss and a BMI change of −0.6 kg/m^2^ compared to a weight gain of +0.7 kg and BMI change of +0.3 kg/m^2^ in the control group. In 2015, Aoike et al^26^ reported the results of an RCT that compared the effects of home-based moderate-intensity aerobic exercise compared to no intervention. Participants in the exercise group lost −0.7 kg of weight with a BMI change of −0.3 kg/m^2^ (+0.2 kg of weight gain and no change in BMI in the group without intervention). Aoike et al^27^ reported the results of an RCT in 2018 in which they examined the effects of home-based aerobic exercise versus center-based aerobic exercise versus usual care. The BMI change was −0.7 kg/m^2^ in the home-based group versus −0.6 kg/m^2^ in the center-based group versus +0.8 kg in the control group. The SLIMM initiative by Lyden et al,^29^ which included feedback and check-ins with participants, changed participant BMI by −1.1 kg/m^2^ compared to the control group. Sheshadri et al^28^ used pedometers to encourage physical activity, and the BMI change was −1.0 kg/m^2^ (*P* < 0.01).

Finally, the combined effects of nutritional intervention with supervised exercise were examined in 5 studies. A prospective cohort study by Malin et al^20^ used a combination of a 500-kcal hypocaloric diet with macronutrient control and supervised aerobic activity. This approach led to a weight loss of −5.3 kg (*P* < 0.001) and a BMI change of −1.9 kg/m^2^ (*P* < 0.001). In a prospective cohort study, Navaneethan et al^32^ prescribed participants a dietary reduction of 500 kcal/d along with supervised aerobic exercise. In this study, the participants had a body weight change of −5.1 kg (*P* = 0.004) and BMI change of −1.9 kg/m^2^ (*P* = 0.004).^32^ An RCT conducted by Patil et al^21^ studied the effects of a 500-1000 kcal/d caloric restriction with moderate-intensity physical activity and noted a BMI change of −1.95 kg/m^2^ (*P* = 0.00). In their RCT, Ikizler et al^19^ observed weight loss in the caloric restriction plus exercise group of 2.39 kg (*P* = 0.02) and a BMI change of −0.83 kg/m^2^ (*P* = 0.03). St-Jules et al^31^ conducted an RCT and investigated the effects of a 500 kcal/d caloric restriction along with 150 min/wk of unsupervised physical activity in conjunction with either educational sessions, social cognitive theory-based group counseling, or technology-based self-monitoring. In this study, all groups had lost weight at 6 months. Participants who underwent combined group counseling and self-monitoring experienced a weight reduction of 2.7 kg, self-monitoring had a weight reduction of 2.3 kg, group counseling lost 1.4 kg, and education alone lost 1.2 kg of weight at the end of the study.^31^

#### Other Measures of Body Composition

Change in body fat percentage was described in 7 studies. The very low-calorie diet in Friedman’s prospective cohort study^18^ resulted in a −6.4% (P = 0.11) loss. Ikizler’s combined exercise and nutrition intervention^19^ demonstrated a decrease of −0.52% in body fat (*P* = 0.02) compared with a −1.58% reduction with the nutritional intervention alone (*P* = 0.02). Malin’s hypocaloric diet with exercise^20^ demonstrated −2.3% body fat reduction (*P* = 0.001). Time-restricted feeding in the prospective cohort study of Lao et al^25^ showed a −1% change in the intervention group versus −0.5% in the control group (*P* = 0.59). Navaneethan et al’s^32^ combined hypocaloric diet and aerobic exercise noted a −1.8% (*P* = 0.004) change in body fat at 3 months.

In terms of exercise alone, Aoike et al^27^ demonstrated an insignificant effect on body fat (+0.6% in the home-based aerobic activity versus −0.1% in the control group). Lyden’s RCT demonstrated a between-group difference in percentage body fat loss of −2.2% with the SLIMM program.^29^

The effect of nutritional intervention and exercise on waist circumference was described in 5 studies. Navaneethan et al’s^32^ combined hypocaloric diet and aerobic exercised described a waist circumference reduction of −3.0 cm (*P* = 0.004). In Lao et al’s^25^ prospective cohort study, time-restricted feeding appeared to reduce waist circumference by −1.9 cm compared to +0.1 cm in the control group (*P* = 0.110). In their RCT, Howden et al^30^ investigated the effects of combined aerobic exercise and resistance training and saw a reduction of −1.4 cm in the intervention compared to +0.6 cm in the control group (*P* = 0.01). In Lyden et al,^29^ SLIMM decreased waist circumference by −0.4 cm. Finally, waist-to-hip ratio was described in the RCT by Patil et al,^21^ in which the mean waist-to-hip ratio decreased by 0.03 cm after 6 months of a hypocaloric diet with exercise (*P* < 0.001).

#### Other Outcomes

All the studies except 3 had follow-up rates over 80%. The study with the lowest retention rate was Pereira et al’s^24^ prospective cohort study of CBT and dietary coaching, which had a completion rate of 69% (23/33). In this study, 4 patients left because of health reasons. St-Jules et al’s^31^ study on combined nutritional intervention, exercise, education, and counseling had a completion rate of 73% (186/256) with fewer dropouts in groups with counseling or technology-based self-monitoring. Navaneethan et al^32^ had a completion rate of 75% (9/12). In this study, one of the participants dropped out because of pre-existing back pain that was worsened during the study. No study commented on significant adverse events, such as new injuries or malnutrition.

## Discussion

Among individuals with CKD and obesity, nonpharmacologic and nonsurgical weight loss can be achieved with caloric restriction and exercise in combination with multidisciplinary support. However, the literature was limited. Only 7 studies were RCTs. There was considerable heterogeneity in the amount of weight loss and the change in BMI and body composition. Studies included participants at varying stages of CKD, but dialysis patients were studied less often.

The greatest amount of weight loss was achieved in the studies that used very low-energy diets with or without exercise. However, the duration of these studies was short. Previous studies suggest that very low-calorie diets are associated with weight regain after completing the diet.^34^ This may be because of increases in appetite; a study by Polidori et al^35^ suggested that for each kilogram of weight loss, there is a proportional increase in appetite above baseline by about 100 kcal/d. Although very low-energy diets may be effective in the short term, it would be very important to understand if they are sufficient to sustain weight loss over the longer term. From this review, it is also unclear if restricting certain caloric sources, such as fats or carbohydrates, is beneficial for people with CKD.^36^

In the general population, there has been growing interest in time-restricted eating for weight management, but only 1 study focused on people with CKD. This study not only used time restriction in eating but also encouraged a high-quality low-protein diet for the intervention arm. It is unclear if time-restricted eating or the macronutrient composition of the diet resulted in the significant weight loss or changes in body composition.

It is important to note that 15 studies involved multidisciplinary health care professionals in the weight loss intervention by directly monitoring progress, coaching, and encouraging compliance. Significant changes in body weight or body composition measures were seen in 4 of the 7 studies. This highlights the importance of including dieticians and allied health team members in developing the interventions. Optimal diets for people with kidney disease are restrictive and often conflict with recommended food plans for other, and often coinciding, conditions (ie, diabetes). This places an additional burden on people, and additional coaching may be required.^37^ Contemporary tools such as mobile or electronic health applications have been shown to be effective in helping people manage chronic diseases such as diabetes, chronic lung disease, and cardiovascular disease.^38^^,^^39^ The only study that used technology-based self-monitoring was St-Jules et al,^31^ which showed some efficacy in sustained weight loss.

Personalized coaching and management plans may also be important to achieving results, maintaining compliance, and sustaining long-term weight loss. Two studies showed significant changes in body composition measures. Additionally, the intervention arms in both studies had over 80% completion rate. This is congruent with expert consensus in Obesity Canada’s 2020 practice guidelines for Primary Health in Obesity Management.^40^ Thus, incorporating motivational coaching into a weight loss intervention may enhance body composition changes and overall adherence.

The results of this review may complement recently published studies on pharmacologic therapy in patients with CKD. The FLOW trial demonstrated the glucagon-like peptide-1 receptor agonist (semaglutide) is safe to use in patients with high risk of CKD and estimated glomerular filtration rates as low as 25 mL/min/1.73 m^2^. In this trial, the participants in the semaglutide arm lost more weight than the placebo arm (−5.55 kg in the intervention vs −1.45 kg in the placebo with an estimated difference of −4.1 kg; 95% CI, −4.56 to −3.65 kg).^13^ There were no specified dietary or exercise suggestions for participants or any described coaching or multidisciplinary involvement. One wonders if there may have been more weight loss if semaglutide was combined with nonpharmacologic interventions.

It is also important to highlight that none of the studies included participants as partners in the design or implementation of their interventions. Engaging participants as partners may improve research design and even potentially improve compliance.^41^ This may be important when investigating outcomes, such as weight loss, which require both patient retention to the study as well as compliance with treatment protocols.

We conducted a thorough review of the medical literature on weight loss interventions using a librarian-developed, broad search strategy. This review focused on an understudied population, typically excluded from research studies. Limitations are that obesity was typically defined by BMI, which may not be the most accurate measure of adiposity in this population. However, it is the most readily available anthropometric measure used for clinical decision making and research studies. Additionally, we only included articles in English and did not include gray literature, which might have omitted some relevant studies.

Our review is also limited by the heterogeneric population of the studies included; some lacked details including the breakdown of participants by estimated glomerular filtration rate and/or BMI. Therefore, our study may be over-inclusive with some people without severe CKD or BMI ≥30 kg/m^2^. Studies also did not consistently examine adverse events from weight loss interventions (ie, malnutrition, new musculoskeletal injuries). This is important because in a recent study of adults with CKD and obesity, BMI loss with concomitant serum albumin levels or fat-free mass loss was associated with a high risk of death.^42^

In people with advanced CKD and obesity, the combination of personalized dietary and physical activity interventions and the use of a multidisciplinary team appear to facilitate modest weight loss even without pharmacotherapy. Integrating nonpharmacologic and nonsurgical therapy is essential for an evidence-based and patient-centered weight management intervention to address obesity as a barrier to kidney transplant.
